# Evaluating the impact of boxing on prefrontal cortex activation and cognitive performance: A pilot study using fNIRS technology and the Stroop test

**DOI:** 10.1371/journal.pone.0314979

**Published:** 2024-12-13

**Authors:** Muhammed Sıddık Çemç, Fatih Ağduman

**Affiliations:** 1 Department of Physical Education and Sports, Boğaziçi University, Istanbul, Türkiye; 2 Department of Recreation, Faculty of Sport Sciences, Atatürk University, Erzurum, Türkiye; 3 Sport Sciences Research and Application Center, Atatürk University, Erzurum, Türkiye; Virginia Tech: Virginia Polytechnic Institute and State University, UNITED STATES OF AMERICA

## Abstract

This research sets out to investigate the differences in hemoglobin concentration occurring in the prefrontal cortex (PFC) during the administration of the Stroop test in active amateur boxers and to compare the obtained data regarding chronic traumatic brain injury with those of healthy individuals. The research was conducted at the Atatürk University Neuropsychology Laboratory. Participants consisted of 6 male boxers, aged 19.66 ± 2.94 years, who had been actively boxing for 7.5 ± 3.8 years and had received at least high school level education, with right-hand dominance, and 8 healthy males, aged 19.62 ± 1.18 years, who had not engaged in any combat sports. fNIRS recordings were taken over the Prefrontal Cortex (PFC) while Stroop test stimuli were presented to the participants in a block design. The data were analyzed using the JASP program. Mann-Whitney U test was applied to evaluate the differences between groups in Stroop test data. The activation levels on the prefrontal cortex during the test were evaluated using the Repeated Measures ANOVA test. A significance level of p <0.05 was accepted for the analyses. In conclusion, compared to the control group, boxers demonstrated a significantly higher level of cerebral activation in the right dlPFC/vlPFC regions during the congruent task and in the right dmPFC as well as the left dmPFC/vmPFC/OFC regions during the incongruent task in the Stroop test. When the Stroop test results of the participants were evaluated between groups, it was found that although statistically insignificant compared to healthy subjects, boxers generally exhibited failure. In conclusion, it was found that boxers exhibit higher neural activation responses and lower cognitive performance during neurophysiological testing compared to healthy controls. These two conditions are thought to be interconnected and are considered to result from neural inefficiency.

## Introduction

Traumatic brain injury (TBI) is defined as a head injury that can result in a temporary or permanent loss of consciousness or alteration of mental state, sometimes leading to persistent or progressive symptoms. This condition often leads to persistent or evolving symptoms that can impact an individual’s standard of living [1]. Epidemiological data from the Centers for Disease Control and Prevention indicate that around 1.7 million individuals in the US sustain a TBI annually, underscoring its significance as a public health concern [2]. The etiology of TBI varies globally, with falls and motor vehicle collisions accounting for the majority of cases in both developing and developed nations. Incidence rates exhibit demographic variability, with infants under one year primarily affected by hypoxia or anoxia, children and the elderly most susceptible to falls from heights, adolescents and young adults to sports injuries, and adults to motor vehicle accidents. These findings highlight the need for targeted prevention strategies across different age groups [3].

In recent years, sports-related traumatic brain injury (TBI) has gained significant attention in the scientific literature. Some studies have identified neurological, neuropsychological, and cognitive impairments such as depression, cognitive dysfunction, early-onset Alzheimer’s disease, and dementia in amateur and professional athletes due to repetitive head impacts [4]. In athletes, symptoms of chronic traumatic encephalopathy (CTE) are reported to manifest years after repeated head impacts, typically emerging in middle age or later. The onset of damage is influenced by factors such as the frequency, severity, and duration of the impacts sustained. Although no specific number of impacts is identified, repeated head trauma in athletes accumulates over the years, leading to neurodegenerative processes. In particular, frequent head injuries in contact sports significantly increase the risk of developing CTE [5].

The growing popularity of boxing and other combat sports is attributed to their recognized benefits in enhancing physical fitness and providing skills for personal defense [6, 7]. Despite these advantages, participation in these sports carries the risk of sustaining various injuries, including those that can be fatal [8]. Given the nature of boxing, where the head often becomes the primary focus of an opponent’s attack, practitioners are particularly vulnerable to both immediate and prolonged neurological harm [9]. The British Medical Association (BMA) has steadfastly called for a ban on boxing due to the accumulating evidence of both immediate and long-term injuries related to the sport. Citing concerns over cumulative brain damage, also known as chronic traumatic brain injury, the BMA’s publications have repeatedly called for a comprehensive ban on both amateur and professional boxing [10, 11].

Boxing, like other sports, is driven by the goal of victory, often through the explicit intention to inflict harm upon the opponent, which inherently increases the risk of injury. This sport distinctively prioritizes strikes to the head, aiming to diminish the opponent’s defensive capabilities or to induce injuries severe enough to compel a referee to halt the match, thereby securing a win [12]. This strategy elevates boxing’s risk profile significantly when compared to other sports, with a notably higher incidence of fatalities and acute traumatic brain injuries (TBI) relative to participant numbers [13]. The nature of boxing, which fundamentally seeks to incapacitate the opponent, naturally predisposes participants to a heightened risk of acute TBI during both matches and training sessions [14].

Neuroscientists employ a variety of neuroimaging techniques to investigate brain function, among which Functional Near-Infrared Spectroscopy (fNIRS) stands out as a non-invasive approach. This method was introduced for applications regarding brain biophysics, psychophysiology, and clinical research, offering a unique insight into cerebral dynamics [15, 16]. fNIRS technology leverages infrared light to probe the prefrontal cortex, enabling the detection and measurement of hemoglobin (Hb), oxygenated hemoglobin (HbO_2_), and deoxyhemoglobin (deoxyHb) concentrations. This capability facilitates a direct assessment of cerebral blood flow and oxygenation, reflecting neural activity in the monitored regions [17].

The application of Functional Near-Infrared Spectroscopy (fNIRS) within the field of neuroscience has seen a substantial increase, as evidenced by its growing utilization in a diverse array of studies [18]. Research employing fNIRS has spanned various domains, including analyses of resting-state dynamics, visual and linguistic processing, attention and memory functions, emotional responses, executive functions, somatosensory activities, and motor functions [19]. Functional near-infrared spectroscopy (fNIRS) is currently used to investigate neural information processes related to neurological disorders such as mild cognitive impairment [20, 21], Alzheimer’s disease [22], Parkinson’s disease [23], stroke [24], traumatic brain injury [25], as well as psychiatric conditions such as schizophrenia [26] and anxiety [27].

Neuropsychological assessments have proven to be efficacious in identifying cognitive deficits attributed to participation in contact and collision sports, with research demonstrating their sensitivity in detecting such impairments [28–30]. Among these assessments, the Stroop test, developed by J.R. Stroop in 1935, stands out as a pivotal tool for evaluating selective attention, particularly in individuals who experience sustained TBI [31]. The application of the Stroop test has garnered widespread endorsement for its utility in probing the functionalities of the frontal lobe, a brain region integral to cognitive processing and executive functions [32, 33]. The test intricately examines three cognitive processes: reading, color naming, and selective attention [34], making it an invaluable instrument in the comprehensive assessment of attentional capacities [35].

The current body of literature indicates a gap in neurobiological research concerning the long-term effects of combat sports, particularly boxing, on chronic traumatic brain injury (CTBI). This study is designed to contribute by working on variations in hemoglobin concentration within the Prefrontal Cortex (PFC) during the execution of the Stroop test among active amateur boxers. The objective is to compare these findings with data from healthy control subjects to assess the presence and extent of chronic TBI in the population. With this strategy, the study aims to provide treasured insights regarding the neurobiological impacts of boxing, enhancing our understanding of CTBI in athletes engaged in high-risk sports.

## Materials and methods

All measurements of this study were conducted per the latest version of the Helsinki Declaration at the Neuropsychology Laboratory of the Atatürk University Center for Sport Sciences Research and Application. Data collection was conducted between March 5 and March 25, 2024. Before measurements, approval from the “Ethics Committee of Ataturk University Faculty of Sport Sciences” was granted for the present research (Number: E-70400699-000-2300340168, Dated: 25.10.2023).

### Participants

The research involved fourteen right-handed participants, all of whom had attained at least a high school level of education. The experimental group consisted of six male boxers, with an average age of 19.66 ± 2.94 years, who had been actively engaged in boxing for an average duration of 7.5 ± 3.88 years. The control group comprised eight healthy males with an average age of 19.62 ± 1.18 years and no history of participation in combat sports.

### Study design

Before the implementation of the study, participants were thoroughly briefed on the measurement device and the test procedures. Health screenings were conducted to confirm the absence of any chronic or acute illnesses among participants. They were told not to use alcohol, tobacco, and caffeine for 24 hours prior to undergoing the test and measurement. The environmental conditions for the test were meticulously controlled, set at a temperature of 21°C, with a noise level of 15 dB, optimal humidity, and isolation from electromagnetic fields to prevent any external interference. All test sessions were conducted under uniform conditions to ensure comparability of data. Participants wore comfortable clothing and were neither hungry nor fatigued during the test, which was scheduled at noon to accommodate their biological clocks. The timing for the test was standardized, providing equal opportunity for all participants. Data collection was efficiently completed within one day, requiring only one session per participant.

### Data collection

#### Edinburgh Inventory Hand Preference Survey

For assessing participants’ dominant hand usage, the "Edinburgh Inventory Hand Preference Survey" [36] was administered. Participants answered survey questions reflecting their hand usage, and responses were evaluated using the Geschwind score. Total scores determined each participant’s hand preference as left-handed, ambidextrous, or right-handed.

#### fNIRS measurements

Throughout the stimulus presentation phase, Functional Near-Infrared Spectroscopy (fNIRS) technology was used to track variations in hemoglobin levels across the prefrontal cortex of the subjects. fNIRS measurements were obtained by placing a cap/headgear on participants’ heads, utilizing the NIRSport2 device (NIRx Medizintechnik GmbH, Germany) equipped with 16 optodes (8 sources and 8 detectors). The optodes were positioned over the prefrontal cortex in accordance with the experimental objectives, and recordings were conducted in the 760–850 nm range using continuous wave (CW) methodology. The Color-Word Stroop Test (CWST) compatible stimuli, both congruent and incongruent, were presented to participants in a block design through the Psychtoolbox for MATLAB 2018a program.

Within the scope of the study, changes in oxygenated hemoglobin (HbO_2_) in the cerebral cortex of participants were detected during the stimulus presentation. The analysis aimed to identify the difference between the neurophysiological state during stimulus presentation (quantity of oxygenated hemoglobin) and the baseline state by subtracting the amount of baseline oxygenated hemoglobin.

After placing the optodes on participants’ heads while they were in a seated position, recordings were conducted during the administration of the Stroop test using computer assistance. The process of cap and optode placement, as well as the experimental session, lasted approximately 15 minutes. Data obtained from the fNIRS device were integrated with behavioral data for subsequent analysis. The cap used in the study conforms to the MNI–Montreal neurological institute standards and is placed on the participants’ heads after the measurement of the distance between the inion and nasion areas.

**Fig 1:**
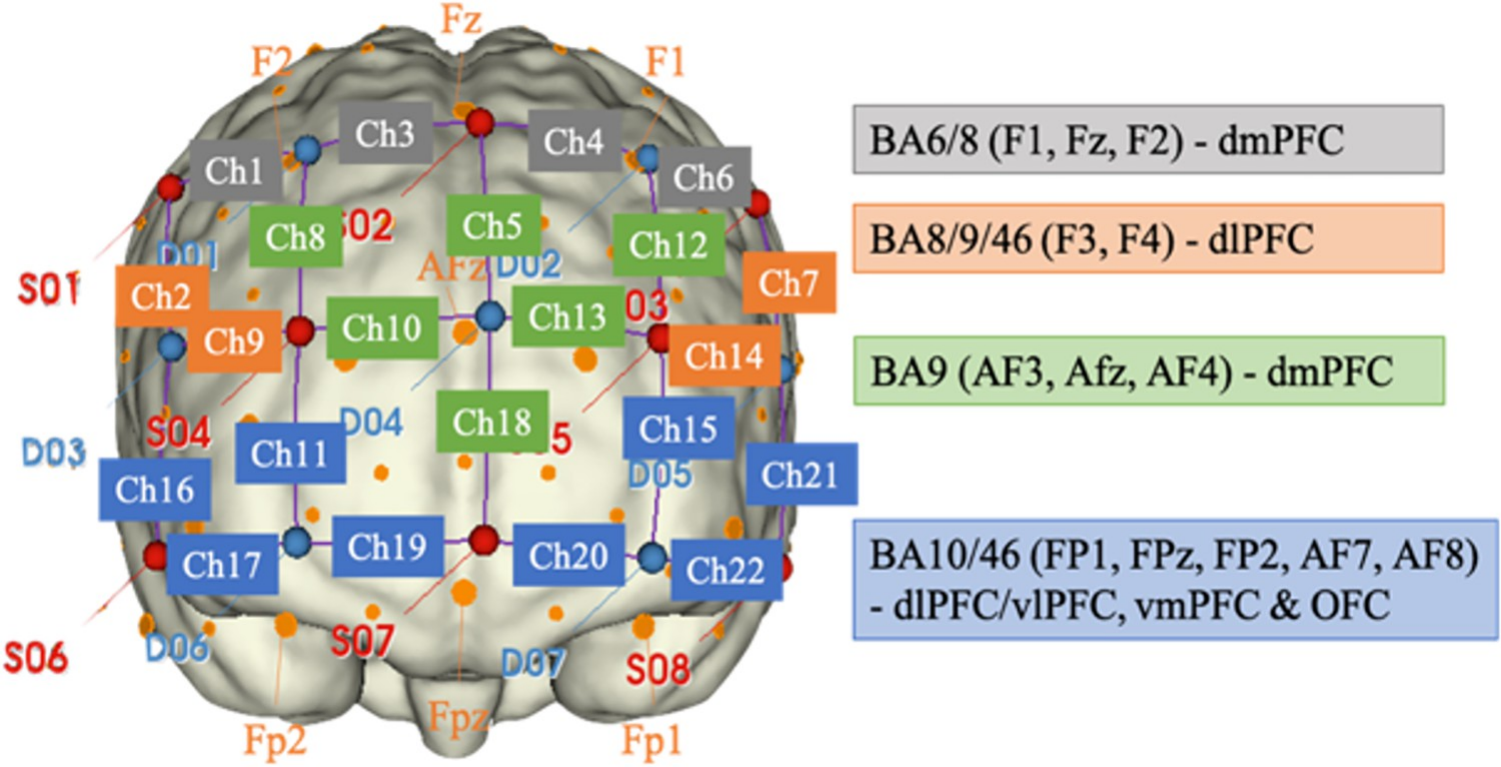
fNIRS measurement assembly plan and brain regions where channels record.

Fig 1 illustrates the fNIRS measurement assembly plan and the corresponding brain regions from which the channels are recorded [37]. The fNIRS data was processed using a Principal Component Analysis (PCA) filter applied through scripts developed in MATLAB software, and the data captured between 5–25 seconds was analyzed under the Contrast-to-Noise Ratio (CNR) method. The CNR matrix operation involves calculating the baseline average of 60 seconds at the beginning and end of the test, subtracting the highest HbO_2_ value formed within the 5 to 25-second interval, and finally terminating the operation by dividing the resultant value by the HbO_2_ standard deviation that occurred over the baseline.


$$The\;Contrast-to-Noise\;Ration\;(CNR)\;matrix=\frac{\left(max(y(act5))-mean(base)\right)}{std(y(base))}$$

Quantities of oxyhemoglobin measured in the prefrontal cortex of the brain were recorded at a frequency rate of 10.1725 Hz. After the calculation of oxyhemoglobin quantities, a Butterworth low-pass filter with a cutoff frequency of 0.25 Hz was applied to eliminate baseline deviations and remove fluctuations caused by heart rate, respiration, and similar factors.

#### Stroop test

The Stroop test was coded and generated in the Psychtoolbox for MATLAB 2018a program on a desktop computer with a 64-bit Windows 10 operating system, NVIDIA GeForce 950M graphics card, Intel© i7 - 7500U @ 2.7 GHz processor, and 16 GB RAM.

The Stroop test consists of congruent and incongruent task sessions [38]. In the congruent task, the color displayed at the top was presented in accordance with the text (e.g., "RED" with a red background, "GREEN" with a green background, or "BLUE" with a blue background), and participants were asked to match it with one of the options below. In the incongruent task, colors and text were presented differently, and participants were asked to match the color above with the correct text among the two options below. All words were written in Turkish characters. The stimuli were presented at eye level on a 28-inch LCD monitor positioned approximately 1.5 meters away from the participants.

**Fig 2:**
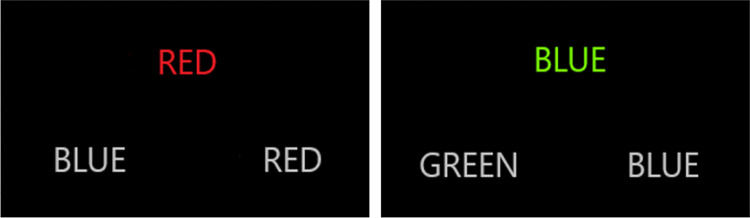
The Stroop task with congruent and incongruent stimuli.

In Fig 2, subjects were directed to provide responses by pressing the "←" and "→" directional buttons with their right index and ring fingers. Before initiating the Stroop test, the content of the test was introduced, and a practice trial (2 min) was conducted by the participants. Following the introduction to the Stroop test, the fNIRS cap was placed on the participants’ heads, and the necessary calibration procedures were performed.

**Fig 3:**
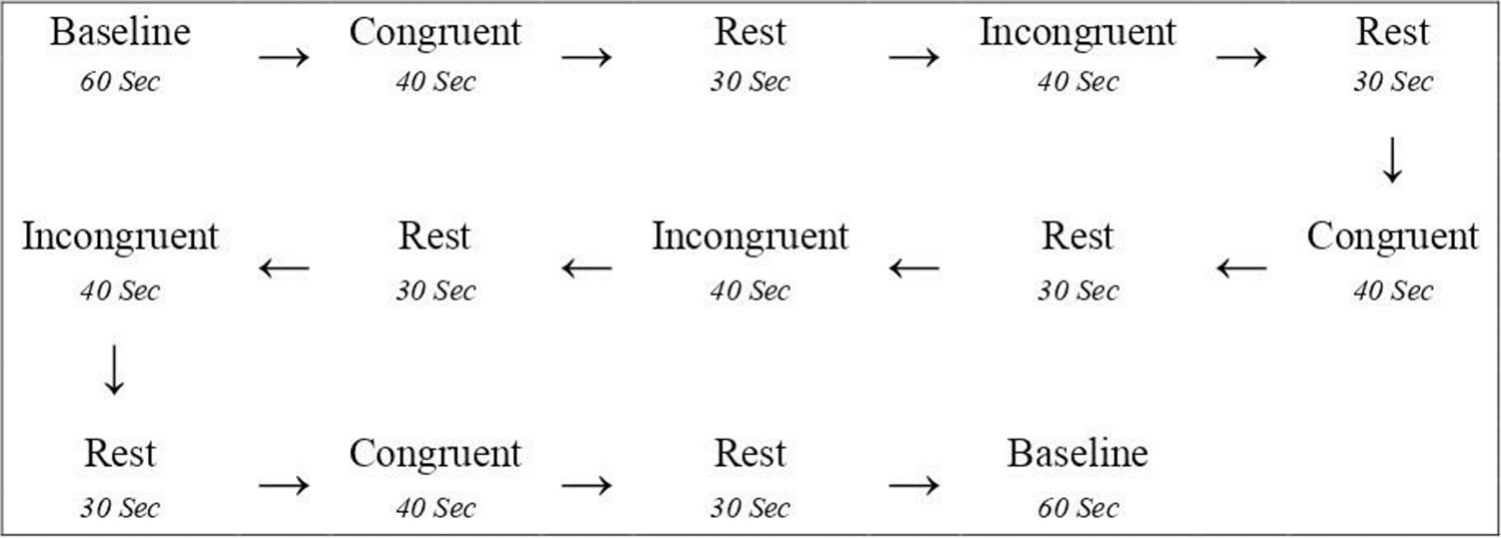
The sequence of the Stroop test paradigm adapted with fNIRS measurement.

The Stroop task, as illustrated in Fig 3, was structured into six sections, composed of three blocks, each with 16 congruent and 16 incongruent stimuli [39], separated by 30-second rest periods [40]. A 60-second baseline was recorded at the beginning and conclusion of the task. Reaction time and error rate were measured. The stimulus continued to be displayed on the monitor until a reaction was elicited or for a duration of 2000 milliseconds. Stimuli appeared every 1000 milliseconds. Responses between 200 to 2000 milliseconds following the appearance of the stimulus were regarded as correct. Responses out of the time range (200–2000 milliseconds) or when the participant selected the incorrect color button were deemed not correct. Following the completion of all participants’ measurements, the data were analyzed.

#### Statistical methods

Standard statistical methods were utilized to compute means and standard deviations in data analysis. The data obtained from the fNIRS recordings were first analyzed using the Shapiro-Wilk test. As a result, it was determined that the data followed a normal distribution [41]. Subsequently, the data were evaluated using Levene’s test, and it was observed that the variances were homogeneous [42].

The normality analysis of the results from the Stroop test’s Congruent and Incongruent tasks was evaluated using the Shapiro-Wilk test. In terms of Congruent Error Rate, both the boxers (w = 0.666, p = 0.003) and the control group (w = 0.566, p < 0.001) showed a significant deviation from normality. For Congruent Reaction Time (Sec), the results indicated that both the boxers (w = 0.887, p = 0.303) and the control group (w = 0.855, p = 0.107) were consistent with normal distribution. Regarding the Incongruent Error Rate, both the boxers (w = 0.781, p = 0.040) and the control group (w = 0.810, p = 0.037) showed a significant deviation from normality. However, for Incongruent Reaction Time (Sec), both the boxers (w = 0.883, p = 0.284) and the control group (w = 0.895, p = 0.258) presented results consistent with normality. These findings suggest that error rates for both the boxers and the control group do not follow a normal distribution. However, in terms of reaction times, both groups demonstrated normal distribution in both congruent and incongruent tasks. Therefore, it is appropriate to use non-parametric tests for the evaluation of error rates between the groups [41].

The data obtained were analyzed using the JASP program (University of Amsterdam, Nieuwe Achtergracht 129B, AMS, NL). The Mann-Whitney U test was applied to evaluate the differences between groups in Stroop test data. Additionally, the effect sizes of the Stroop test data were calculated using the Rank-Biserial Correlation Test. Activation levels on the prefrontal cortex during the test were evaluated using the Repeated Measures ANOVA test. The effect sizes were determined using Cohen’s d. A significance level of p < 0.05 was accepted for the analyses.

## Results

In this section, the results obtained from the research are displayed and supported with figures.

**Fig 4:**
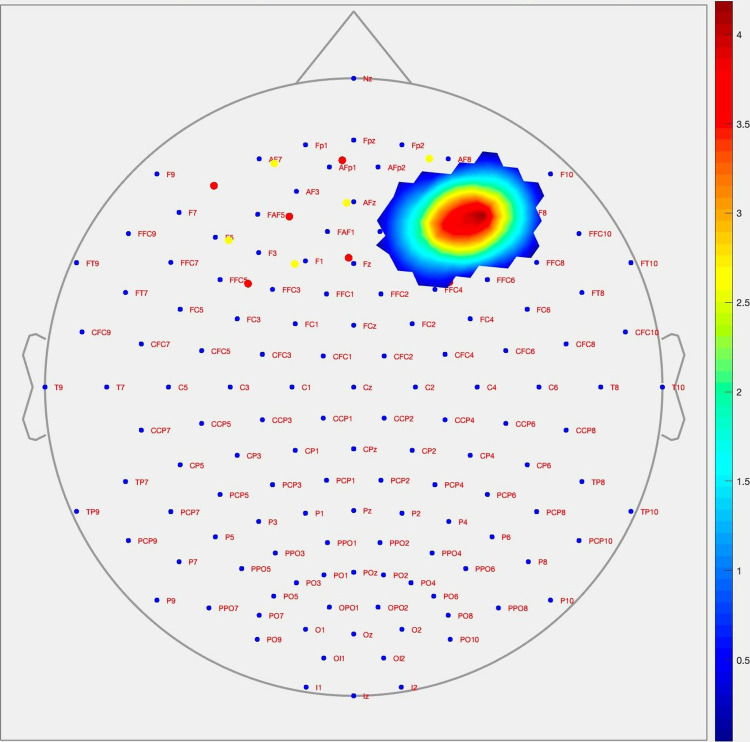
Topographic image of the prefrontal cortex (PFC) derived from the changes in activation level during the Stroop test congruent stimuli between groups of participants using the CNR method.

During the Stroop test congruent stimulus condition, as shown in Fig 4, it was observed that cerebral activation levels in boxers were significantly higher compared to the control group in the right dorsolateral prefrontal cortex (dlPFC) (channel 9; p = 0.009; Cohen’s d = 1.226) and right ventrolateral prefrontal cortex (vlPFC) (channel 16; p = 0.013; Cohen’s d = 1.362) regions.

**Fig 5:**
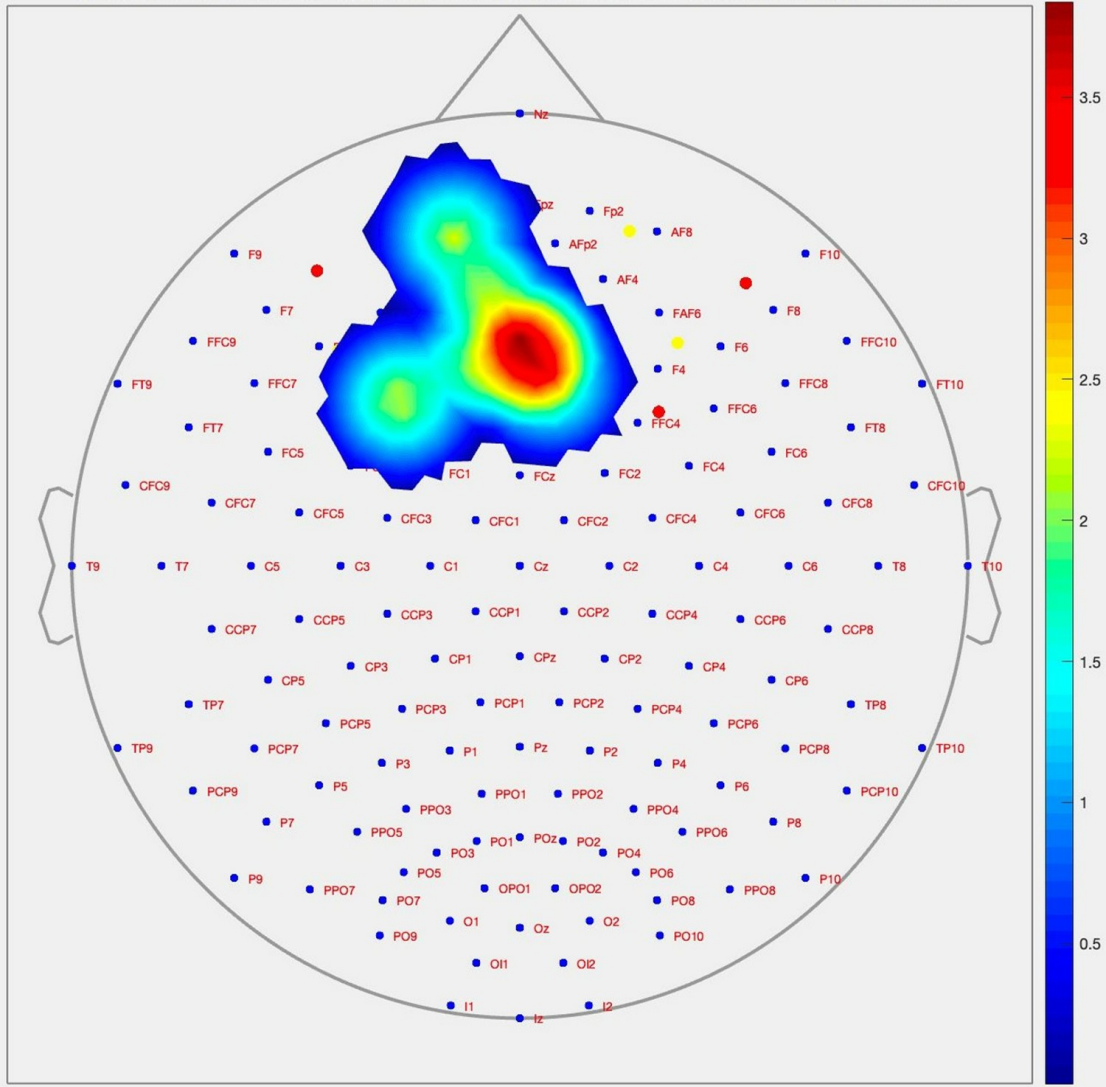
Topographic image of the prefrontal cortex (PFC) derived from the changes in activation level during the Stroop test incongruent stimuli between groups of participants using the CNR method.

During the Stroop test incongruent stimulus condition, as shown in Fig 5, it was observed that cerebral activation levels in boxers were significantly higher compared to the control group in the following regions: right dorsomedial prefrontal cortex (dmPFC) (channel 3; p = 0.022; Cohen’s d = 1.088) (channel 5; p = 0.016; Cohen’s d = 1.162), left dorsomedial prefrontal cortex (dmPFC) (channel 6; p = 0.042; Cohen’s d = 0.872), and left ventromedial prefrontal cortex (vmPFC) and orbitofrontal cortex (OFC) (channel 20; p = 0.023; Cohen’s d = 1.140).

**Fig 6:**
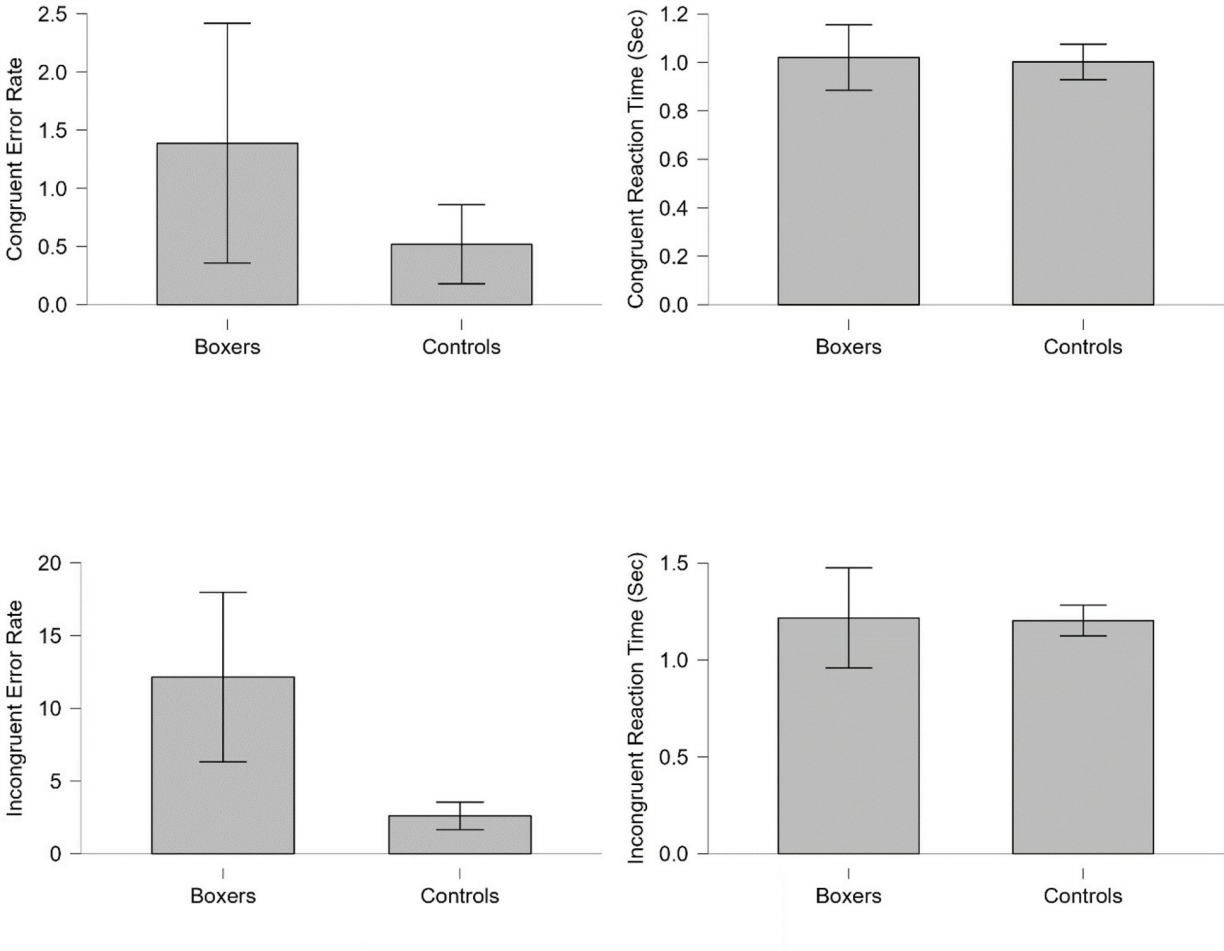
The intergroup analysis graphs of the data obtained within the scope of the Stroop test.

The Fig 6 above illustrates the differences in values obtained by participants in the Stroop test between the groups. According to the statistical analysis conducted, in terms of error rate in the congruent test, boxers reached an error rate of 1.388±1.030, while controls reached 0.520±0.340 error rate. Boxers exhibited a higher error rate compared to the control group. However, this difference observed is not statistically significant (w = 27.000, p = 0.684, r_rb_ = 0.125). When examining reaction times in the congruent test, boxers had a reaction time of 1.020±0.053 seconds, while controls had a reaction time of 1.002±0.031 seconds. The control group showed a lower reaction time compared to boxers. However, this difference observed is not statistically significant (w = 29.000, p = 0.573, r_rb_ = 0.208). Regarding error rate in the incongruent test, boxers reached an error rate of 12.152±5.829, while controls reached 2.603±0.944 error rate. Boxers exhibited a higher error rate compared to the control group. However, this difference observed is not statistically significant (w = 37.500, p = 0.080, r_rb_ = 0.563). When examining reaction times in the incongruent test, boxers had a reaction time of 1.216±0.100 seconds, while controls had a reaction time of 1.202±0.034 seconds. The control group showed a lower reaction time compared to boxers. However, this difference observed is not statistically significant (w = 19.000, p = 0.573, r_rb_ = -0.208).

## Discussion

As a result of this study, amateur boxers demonstrated significantly higher cerebral activation levels compared to the control group during the Stroop test, particularly in the right dlPFC/vlPFC regions during the congruent task and in the right dmPFC and left dmPFC/vmPFC/OFC regions during the incongruent task. When reviewing the existing literature, it has been reported that retired contact sport athletes with a history of concussion exhibit significantly higher levels of oxyhemoglobin (O2Hb) over the left prefrontal cortex during squat and upright standing movements compared to individuals without a history of concussion [43]. Additionally, individuals with traumatic brain injury (TBI) have been shown to experience greater oxygenation changes compared to healthy controls. Symptomatic subjects exhibited more oxygenation changes during the King-Devick (KD) test than asymptomatic subjects, suggesting that symptomatic individuals display a higher cognitive workload model compared to their asymptomatic counterparts [44]. It was also observed that individuals with TBI displayed significantly increased levels of oxyhemoglobin in bilateral frontal regions during the congruent task of the Stroop test compared to healthy controls, with the relatively higher neural activity in the frontal lobes during this simple task suggesting neural inefficiency in the TBI group. Furthermore, during the incongruent task, individuals with TBI made more errors compared to the control group [40]. It has also been found that head trauma experienced during athletic activities leads to significant increases in brain activation in the left middle frontal gyrus [45]. Within the scope of this study, the activation differences observed between the boxer and healthy control participants, as measured through the CNR method, and the persistently higher responses in boxers compared to the control group suggest that these differences may be a result of the punches received during active boxing. In conclusion, the similarity of these findings to results from studies on individuals with traumatic brain injury in the literature further strengthens this hypothesis.

Contrary to the findings mentioned above, individuals who experienced sport-related concussions within a 15-45-day period showed decreased brain activation during word memory, design memory, symbol matching, and working memory tasks compared to the healthy control group [46]. In individuals with head trauma, it has also been reported that there is lower consistency in oxyhemoglobin levels over the right hemisphere during resting state and the finger-tapping test compared to healthy individuals [47]. Additionally, it has been found that following head trauma, there were no significant differences in cerebral hemodynamics during light, moderate, and high-intensity exercises [48].

When comparing the results of the Stroop test between groups, it was found that boxers had higher error rates in both the congruent and incongruent tasks compared to the control group, though this difference was not statistically significant. Regarding reaction times for the congruent and incongruent tasks, the control group exhibited shorter reaction times than the boxers, but this difference was also not statistically significant. A review of the literature shows that athletes with concussions performed significantly worse than controls during tests 48 hours post-concussion [49], and athletes who suffered a concussion demonstrated significantly delayed reaction times in the incongruent Stroop task compared to healthy controls [50]. Rugby players, when compared to athletes in non-contact sports, showed significantly lower error rates and reaction times in the Stroop test [51]. Athletes with multiple concussions performed significantly worse in the Stroop test than those with a single concussion [52], and older adults with a history of head trauma had higher error rates in the incongruent task of the Stroop test compared to healthy individuals [53]. Considering these findings, it is suggested that the observed impairments in cognitive abilities may be the result of frequent head trauma over an extended period. In boxing, the force of punches to the head can cause significant damage to opponents. Although no statistically significant difference in Stroop test performance was found between the boxers and healthy individuals in this study, it was generally observed that the boxers underperformed compared to the control group in both the congruent task (error rate and reaction time) and the incongruent task (error rate and reaction time). These findings are believed to be linked to the participants’ boxing experience.

## Conclusion

In conclusion, it was found that boxers exhibited higher neural activation responses during cognitive performance compared to healthy controls, while their neuropsychological test performance was lower, although not statistically significant. These two conditions are thought to be interconnected and may be a result of neural inefficiency. Boxing, by its nature, involves targeting the head with punches. Therefore, to minimize potential damage to human health, it is recommended that athletes perform this sport with the use of protective equipment.

### Limitations and future studies

The study was conducted with 6 active amateur boxers and 8 healthy participants. The fact that all participants were male limits the generalizability of the findings to female boxers or other gender groups. The study focused solely on boxers, without examining athletes from other contact sports, which makes it difficult to generalize the findings to other sports disciplines. Additionally, only the Stroop test was used within the scope of neuropsychological testing to assess cognitive performance.

Given the preliminary nature of this study and the limited sample size, it is suggested that future research replicate these findings with a larger participant group to enhance statistical power and generalizability. Additionally, longitudinal studies would be beneficial to examine how the cognitive and neural effects of boxing evolve over time. The findings from this research may contribute to the development of cognitive training and preventive programs for athletes in contact sports. Specifically, interventions and preventive measures designed to mitigate the long-term effects of repeated head trauma will play a critical role in safeguarding the health of athletes at risk.
